# Expression of Inducible Nitric Oxide Synthase (iNOS) in Microglia of the Developing Quail Retina

**DOI:** 10.1371/journal.pone.0106048

**Published:** 2014-08-29

**Authors:** Ana Sierra, Julio Navascués, Miguel A. Cuadros, Ruth Calvente, David Martín-Oliva, Rosa M. Ferrer-Martín, María Martín-Estebané, María-Carmen Carrasco, José L. Marín-Teva

**Affiliations:** Departamento de Biología Celular, Facultad de Ciencias, Universidad de Granada, Granada, Spain; Hospital Nacional de Parapléjicos – SESCAM, Spain

## Abstract

Inducible nitric oxide synthase (iNOS), which produce large amounts of nitric oxide (NO), is induced in macrophages and microglia in response to inflammatory mediators such as LPS and cytokines. Although iNOS is mainly expressed by microglia that become activated in different pathological and experimental situations, it was recently reported that undifferentiated amoeboid microglia can also express iNOS during normal development. The aim of this study was to investigate the pattern of iNOS expression in microglial cells during normal development and after their activation with LPS by using the quail retina as model. iNOS expression was analyzed by iNOS immunolabeling, western-blot, and RT-PCR. NO production was determined by using DAR-4M AM, a reliable fluorescent indicator of subcellular NO production by iNOS. Embryonic, postnatal, and adult *in situ* quail retinas were used to analyze the pattern of iNOS expression in microglial cells during normal development. iNOS expression and NO production in LPS-treated microglial cells were investigated by an *in vitro* approach based on organotypic cultures of E8 retinas, in which microglial cell behavior is similar to that of the *in situ* retina, as previously demonstrated in our laboratory. We show here that amoeboid microglia in the quail retina express iNOS during normal development. This expression is stronger in microglial cells migrating tangentially in the vitreal part of the retina and is downregulated, albeit maintained, when microglia differentiate and become ramified. LPS treatment of retina explants also induces changes in the morphology of amoeboid microglia compatible with their activation, increasing their lysosomal compartment and upregulating iNOS expression with a concomitant production of NO. Taken together, our findings demonstrate that immature microglial cells express iNOS during normal development, suggesting a certain degree of activation. Furthermore, LPS treatment induces overactivation of amoeboid microglia, resulting in a significant iNOS upregulation.

## Introduction

Microglia are cells responsible for innate cell immunity in the central nervous system (CNS); they have a ramified morphology in the adult CNS and continuously survey the local environment by extending and retracting their highly motile cell processes [1], [2]. Ramified microglia derive from differentiation of amoeboid microglia, which are proliferating and phagocytic amoeboid cells of yolk sac origin that enter the CNS during development and migrate long distances to colonize all CNS regions [3]–[8]. Under pathological conditions in the adult CNS, ramified microglia become activated, retracting their processes and acquiring a macrophage-like rounded morphology [9]–[11] reminiscent of amoeboid microglia during development. Activated microglia upregulate their migratory, proliferative and phagocytic capacities to perform their macrophage-like defensive functions, thereby becoming similar to amoeboid microglia. Activated microglia are able to produce a panoply of neurotoxic and neurotrophic mediators [12]–[16], including nitric oxide (NO).

NO is a liposoluble radical gas that traverses freely across cell membranes and acts as a cellular signaling molecule that participates in various biological effects without the need for a specific membrane receptor. It has an extremely short half-life of only a few seconds due to its rapid reaction with different substances, as a consequence of its unpaired electron [17], [18]. NO has been extensively demonstrated to have a role in neurotoxicity [19]–[26]. However, NO alone is not directly responsible for neuronal apoptosis [27] and can have beneficial effects on cell survival [28], [29]. In fact, it has a pivotal role in regulating signaling pathways involved in neuronal survival in the retina [30], it promotes neuronal protection induced by ischemic preconditioning [31], and it can contribute to endothelial cell survival [32]. Many of the cytotoxic effects of NO appear to be mediated by its oxidation products rather than by NO itself. Thus, simultaneous production of NO and superoxide by activated microglia, under proinflammatory conditions, gives rise to the formation of peroxynitrite, a powerful oxidant that induces neuronal death [18], [27], [28], [33]–[35].

Synthesis of NO from L-arginine and molecular oxygen is catalyzed by NO synthases (NOS) [18], [29], [36]. Three isotypes of these enzymes have been identified: endothelial NOS (eNOS), neuronal NOS (nNOS), and inducible NOS (iNOS). eNOS and nNOS are constitutively expressed in endothelial cells and neurons, respectively, while iNOS is expressed in macrophages, microglia, astrocytes, and other cell types in response to inflammatory mediators such as LPS and cytokines [17], [18], [34].

iNOS appears to be mainly expressed by microglia after their activation by inflammatory factors [13], [34], [37], although some amoeboid microglia were recently reported to express iNOS during the normal development of the CNS [38], [39]. This would be related to the fact that amoeboid microglia undergo a certain degree of activation during normal CNS development, as described in the whole brain [39], the spinal cord [40], and the corpus callosum, fornix, and external capsule [41] of developing mice. In connection with these studies, the present article demonstrates the expression of iNOS in amoeboid microglia of the developing quail retina and its downregulation when microglia differentiate to become ramified.

Although amoeboid microglia show a basal activation in the developing CNS, they appear to be able to increase their activation degree in response to different injurious stimuli [42]–[48] by increasing their phagocytic and proliferative activity [46] and their release of pro-inflammatory cytokines [41], [44], [48]. The bacterial endotoxin lipopolysaccharide (LPS) has been extensively used to induce activation of microglia, with a marked increase in the release of pro-inflammatory molecules and concomitant induction of iNOS expression and NO production [19], [49]–[52]. Hence, the LPS model of microglia activation may be useful to show the ability of amoeboid microglia in the developing retina to increase their activation degree. In the present study, upregulation of iNOS expression was observed in amoeboid microglia of LPS-treated organotypic cultures of quail embryo retina explants, suggesting an increase in the basal activation of amoeboid microglia in the developing retina after LPS treatment.

## Materials and Methods

### Animals

Embryonic, posthatched, and adult quails (Coturnix coturnix japonica) were used in this study. Embryonic and posthatched developing retinas were obtained at the following days of incubation (E) and posthatching (P): E8, E9, E14, E16 and P4. Adult retinas from P60 quails were also studied. Non-cultured retinas were used to examine iNOS expression in developing and adult microglia. Organotypic cultures of explants from E8 quail retinas were also used to study changes in iNOS expression after *in vitro* experimental activation of immature microglia.

Experimental procedures were approved by the Animal Experimentation Ethics Committee of the University of Granada, following the guidelines of the European Union Directive 2010/63/EU on the protection of animals used for scientific purposes.

### *In vitro* culture of E8 retina explants

Explants from E8 retinas were cultured *in vitro* on 30-mm Millicell CM culture plate inserts (Millipore, Billerica, MA; pore size 0.4 µm) according to the method described by Stoppini et al. (1991) [53] with some modifications [54]. Retinas were dissected out into cold Gey's balanced salt solution (Sigma, St. Louis, MO) supplemented with 5 mg/mL glucose (Sigma) and 50 IU-µg/mL penicillin-streptomycin (Invitrogen, Paisley, United Kingdom). After removing the pigment epithelium, square explants (3 mm×3 mm) were isolated from the central area of each retina and then placed on Millicell inserts (Millicell CM, Millipore, Bedford, MA, USA; pore size 0.4 µm), vitreal surface down. Millicell inserts were put in six-well plates containing 1 mL/well culture medium composed of 50% basal medium with Earle's salts, 25% Hank's balanced salt solution, 25% horse serum, 1 mM L-glutamine, 10 IU-µg/mL penicillin-streptomycin (all purchased from Invitrogen), and 5 mg/mL glucose. E8 retina explants were then incubated at 37°C in a humidified atmosphere with 5% CO_2_ for 1 hour *in vitro* (hiv) to 24 hiv (E8+1hiv to E8+24hiv) according to the aim of each experiment.

### LPS-induced activation of microglial cells in cultured retina explants

Microglial activation experiments were performed in E8 retina explants. Microglial cells were activated by treating the explants with 5 µg/mL LPS (*Escherichia coli* OB4:1111, Sigma), which was added to the medium from the beginning of the culture. In each experiment, an explant obtained from the central retina of the right eye of a quail embryo was LPS-treated, and a similar explant from the retina of the left eye of the same embryo was cultured without LPS and served as a control. Non-cultured E9 retinas were used to compare the morphological appearance and the lysosomal compartment of microglial cells between E8+24hiv retina explants and *in situ* retinas at an equivalent developmental age.

### Immunocytochemistry

Microglial cells were identified in cultured retina explants and non-cultured retinas by immunolabeling with the monoclonal antibody QH1 (Developmental Studies Hybridoma Bank [DSHB], University of Iowa, Iowa City, IA), which recognizes all quail hemangioblastic cells except for mature erythrocytes [55], including amoeboid, ramified, and activated microglia [56]. iNOS was identified by immunolabeling with two anti-iNOS polyclonal antibodies from different manufacturers (Abcam, Cambridge, United Kingdom, catalog number ab3523; Thermo Fisher Scientific, Rockford, IL, catalog number PA1-036). These antibodies recognize the mouse iNOS (but not other isoforms of NOS, such as eNOS and nNOS) and show reactivity with the chick iNOS, as described on the antibody data sheet. The monoclonal antibody LEP100 (DSHB), specific to avian species, was used to recognize the microglial cell lysosomal compartment, which is increased in activated microglia [47].

Double QH1/anti-iNOS immunolabeling was carried out on wholemounted non-cultured retinas and cultured retina explants, which were fixed in 4% paraformaldehyde in 0.1 M phosphate buffer for 1 h and permeabilized in 0.01 M phosphate buffered saline (PBS) containing 0.1% Triton X-100 (PBS-Tr) for 4 h. They were subsequently incubated overnight at 4°C in polyclonal anti-iNOS diluted 1∶500 in 1% bovine serum albumin in 0.01 M PBS (BSA-PBS) containing 0.25% Triton X-100 (BSA-PBS-Tr), rinsed in PBS-Tr, and incubated for 4 h at room temperature in one secondary antibody, Alexa Fluor 594-conjugated goat anti-rabbit IgG (Molecular Probes, Eugene, OR). After rinsing in PBS-Tr, wholemounts were incubated overnight at 4°C in the monoclonal antibody QH1 diluted 1∶4 in BSA-PBS, rinsed, and incubated for 4 h at room temperature in the other secondary antibody (Alexa Fluor-488 conjugated goat anti-mouse IgG, Molecular Probes). Both secondary antibodies were diluted 1∶1000 in BSA-PBS-Tr. After further rinsing, wholemounts were coverslipped with Fluoromount G (Southern Biotech, Birmingham, AL) with the vitreal side up.

Cross cryosections of noncultured retinas and cultured retina explants were also used for double QH1/anti-iNOS immunolabeling. Specimens were fixed for 1 h at 4°C in 4% paraformaldehyde in 0.1 M phosphate buffer, thoroughly rinsed in PBS-Tr, cryoprotected overnight at 4°C in 20% sucrose in PBS-Tr, and introduced into 7.5% gelatin and 20% sucrose in PBS-Tr. Solidified blocks containing the specimens were then embedded in OCT compound, frozen in isopentane cooled with liquid nitrogen, and stored at −40°C before sectioning on a Leica CM1850 cryostat; 15 µm-thick cryosections were obtained on Superfrost slides (Menzel-Glasser, Braunschweig, Germany), hydrated in PBS, and treated for double QH1/anti-iNOS immunolabeling following a similar schedule to that described for wholemounts with minor modifications (cryosections were not permeabilized and incubation time in secondary antibodies was reduced to 2.5 h). In addition, cell nuclei were stained in immunolabeled cryosections with the nuclear dye Hoechst 33342 (Sigma).

To guarantee the specificity of the iNOS immunolabeling, single anti-iNOS immunofluorescence was also done on retinal wholemounts and cryosections, checking that the iNOS labeling was similar between single and double immunolabeled specimens. Negative controls omitting the primary antibody were also used.

Double immunofluorescence for LEP100 and QH1 was performed in some cultured E8 retina explants, which were fixed and permeabilized with methanol at −20°C for 10 min, rinsed in PBS-Tr, and incubated in normal goat serum (NGS) diluted 1∶10 in BSA-PBS-Tr for 1 h at room temperature. Next, they were incubated in LEP100 antibody (dilution 1∶1 in BSA-PBS-Tr) for 72 h at 4°C, and then in the first secondary antibody (Alexa Fluor 594-conjugated goat anti-mouse IgG diluted 1∶1000 in BSA-PBS-Tr) for 4 h at room temperature. After washing overnight in PBS-Tr and blocking with NGS for 1 h, explants were incubated in QH1 antibody (dilution 1∶4 in BSA-PBS-Tr) for 24 h at 4°C, and then in the second secondary antibody (Alexa Fluor 488-conjugated goat anti-mouse IgG diluted 1∶1000 in BSA-PBS-Tr) for 4 h at room temperature. After washing, retina explants were mounted on slides and coverslipped with Fluoromount.

### Microscopy

Observations of fluorescent specimens were made with a Leica TCS-SP5 confocal microscope (Leica, Wetzlar, Germany). Stacks of confocal optical sections of selected microscopic fields were collected at 0.5–1 µm intervals and projection images were obtained, stored in TIFF format, and digitally prepared with Adobe Photoshop (Adobe Systems, San José, CA).

### Quantification of anti-iNOS immunofluorescence intensity in microglial cells

The anti-iNOS immunolabeling was quantified by measuring the fluorescence intensity in each microglial cell. The fluorescence intensity of the anti-iNOS labeling was measured on confocal images of double QH1/anti-iNOS immunolabeled whole-mounted retinas of E8, E9, E14, P4, and adult quails. The quantitative analysis was made separately in the nerve fiber layer (NFL), inner plexiform layer (IPL), and outer plexiform layer (OPL) except for E8 and E9, when no microglia were present in the IPL and OPL. 30 QH1-positive microglial cells were randomly selected for each age and retinal layer. Once the profile of each cell was obtained in the QH1 channel, the anti-iNOS channel was converted to grayscale, and the average intensity of pixels per microglial cell was measured in this channel by using Image J 1.48i software (NIH, USA). Pixel intensities ranged from 0 to 255 from the darkest to the lightest shade, respectively. Mean anti-iNOS fluorescence intensity per microglial cell was then obtained for each age and retinal layer.

### Quantitative analysis of morphological features of microglial cells

QH1-labeled microglial cells were morphometrically analyzed in LPS-treated and control E8 retina explants and in E9 non-cultured retinas using Image Tool 2.0 software (University of Texas Health Science Center, San Antonio, TX). Cell profile area, cell elongation index, and cell rounding index were determined as indicators of cell morphology to assess changes in the microglial phenotype compatible with microglial activation after LPS treatment. The elongation index of a cell was defined as the ratio of its major axis length to its minor axis length. The cell rounding index was calculated by the formula 4πA/P^2^, where A is the cell profile area in µm^2^ and P is the cell perimeter in µm. The mean values of these cell parameters were determined in 10 LPS-treated E8+24hiv retina explants, 10 control E8+24hiv retina explants, and 10 non-cultured E9 retinas, based on morphometric analysis data in three different square microscopic fields of 0.25 mm^2^ (500×500 µm) in each specimen.

The relative area of the lysosomal compartment profile with respect to the microglial cell profile area was determined in double LEP100/QH1 immunolabeled retina explants. An increase in this cell parameter was considered an indicator of microglial activation. Three square microscopic fields (250×250 µm) were selected in each explant, measuring the profile area of all microglial (QH1-positive) cells in each field and the profile area of lysosomal (LEP100-positive) compartments within microglial cells. The relative area of the lysosomal compartment profile (percentage of the cell profile area occupied by this compartment) was assessed in each field, determining the mean values in 15 LPS-treated E8+24hiv retina explants, 15 control E8+24hiv retina explants, and 15 non-cultured E9 retinas.

### Determination of iNOS protein expression by western blot analysis

Western blot analysis was used to determine the expression of the iNOS protein in LPS-treated and non-treated control E8+12hiv retina explants. Retina explants were rinsed in PBS and centrifuged at 1,600 rpm for 3 min at 4°C. After removing PBS, explants were resuspended in 100 µL lysis buffer (50 mM Tris HCl, pH 8.0, 0.1 mM EDTA, 0.5% Triton X-100, 12.5 mM β-2-mercaptoethanol) containing 1X protease inhibitor cocktail (Roche Applied Science, Indianapolis, IN) for 45 min on ice with shaking and centrifuged at 13,200 rpm for 15 minutes at 4°C. After protein quantification (Bio-Rad Protein Assay, Bio-Rad, Hercules, CA) of the supernatant, 6X SDS reducing buffer (50 mM Tris-HCl pH 6.8, 6 M urea, 6% β-2-mercaptoethanol, 3% SDS, and 0.003% bromophenol blue) was added, and Western blot analysis was carried out using standard procedures. Briefly, 70 µg protein was loaded into each well of a 7.5% SDS-polyacrylamide gel, which was then run in a mini gel system (Bio-Rad), transferring proteins onto a polyvinylidene difluoride membrane (Immun-Blot PVDF Membrane; Bio-Rad) using a Trans-Blot semi-dry electrophoretic transfer system (Bio-Rad). Blots were blocked with 5% milk powder and 0.1% Tween-20 in PBS for 30 minutes and incubated overnight at 4°C with iNOS antibody (Abcam) diluted 1∶500 in blocking solution. After rinsing, blots were incubated for 2 h at room temperature with peroxidase-conjugated anti-rabbit IgG (Sigma) diluted 1∶1000. Antibody reaction was revealed by chemiluminescence using Immobilon Western HRP substrate (Millipore, Billerica, MA, USA) and ChemiDoc-It Imaging System (UVP, Upland, CA). Anti-β-tubulin antibody was used as loading control for normalization of protein levels.

### Analysis of iNOS mRNA expression

Total RNA was extracted from E8, E9, E14, E16, P4, and adult non-cultured retinas and from LPS-treated and control E8+12hiv retina explants using Trizol (Invitrogen, Carlsbad, CA). Briefly, 1 µg RNA from each specimen was used to remove genomic DNA and synthesize cDNA with a QuantiTect Reverse Transcription kit (QIAGEN GmbH, Hilden, Germany) as per manufacturer's instructions. iNOS gene expression was quantitated by real-time PCR (RT-PCR) analysis using the QuantiTect SYBR Green PCR kit (QIAGEN) in the iCycler iQ detection system (Bio-Rad), amplifying 1 µL cDNA in a 20 µL reaction mixture containing 10 µl SYBR Green Master Mix 2x and 2 µl primer (Gg_NOS2_1_SG, QuantiTect primer Assay, QIAGEN). The expression of 18S rRNA (Mn-Rn18s_2_SG, QuantiTect Primer Assay, QIAGEN) was used as an endogenous control. RT-PCR conditions for amplification of iNOS and 18S rRNA genes were 40 cycles, which consisted of denaturation (95°C, 15 s), annealing (55°C, 30 s) and elongation (72°C, 30 s). In the data analysis, sample amplification curves were represented in triplicate for both the iNOS gene and endogenous control gene, determining the cycle threshold (CT) in each case. The 2^−ΔΔCt^ method [57] was used to calculate differences (fold changes) in the expression of iNOS gene between E8, E9, E14, E16, P4, and adult non-cultured retinas, as well as between LPS-treated and non-treated retina explants. iNOS RT-PCR products were run on 1.5% agarose gel, and the bands were digitalized with the PhotoDoc-It imaging system (UVP) and referred to the corresponding 18S rRNA bands.

### Monitoring NO production in microglial cells of cultured retina explants

NO production by microglial cells was monitored in experiments with LPS-treated and control E8+12hiv retina explants by using the membrane-permeable fluorophore diaminorhodamine-4M acetoxymethyl ester (DAR-4M AM, Calbiochem, Darmstadt, Germany), which is a fluorescent indicator sensitive to the NO presence [58], [59]. E8 retina explants cultured for 12 hiv in the presence (5 µg/mL) or absence of LPS, as described above, were treated with 5 µM DAR-4M AM and 0.4 µg/ml Alexa Fluor-488 conjugated QH1 antibody (AF488-QH1) for 1 h at 37°C. AF488-QH1 marked microglial cells in the explants with strong green fluorescent labeling without affecting their physiological behavior [54], whereas DAR-4M AM labeled NO-producing cells with red fluorescence. After AF488-QH1 and DAR-4M AM treatment, the explants were fixed in 4% paraformaldehyde in 0.1 M phosphate buffer for 45 min and coverslipped with Fluoromount G. Observations of wholemounted explants were made using a Zeiss Axiophot microscope (Zeiss, Oberkochen, Germany) equipped for epifluorescence, obtaining micrographs with a Zeiss AxioCam digital camera.

### Statistical analysis

Data are reported as means ± standard error of the mean (SEM). Statistical differences were determined by one-way analysis of variance (ANOVA) followed by Tukey test for multiple comparisons. All analyses were performed using IBM SPSS statistics software version 20.0.0 for Windows (Chicago, IL, USA). Differences were considered significant at P<0.05.

## Results

### iNOS immunolabeling of amoeboid microglia migrating tangentially in the vitreal part of the quail embryo retina

**Figure 1:**
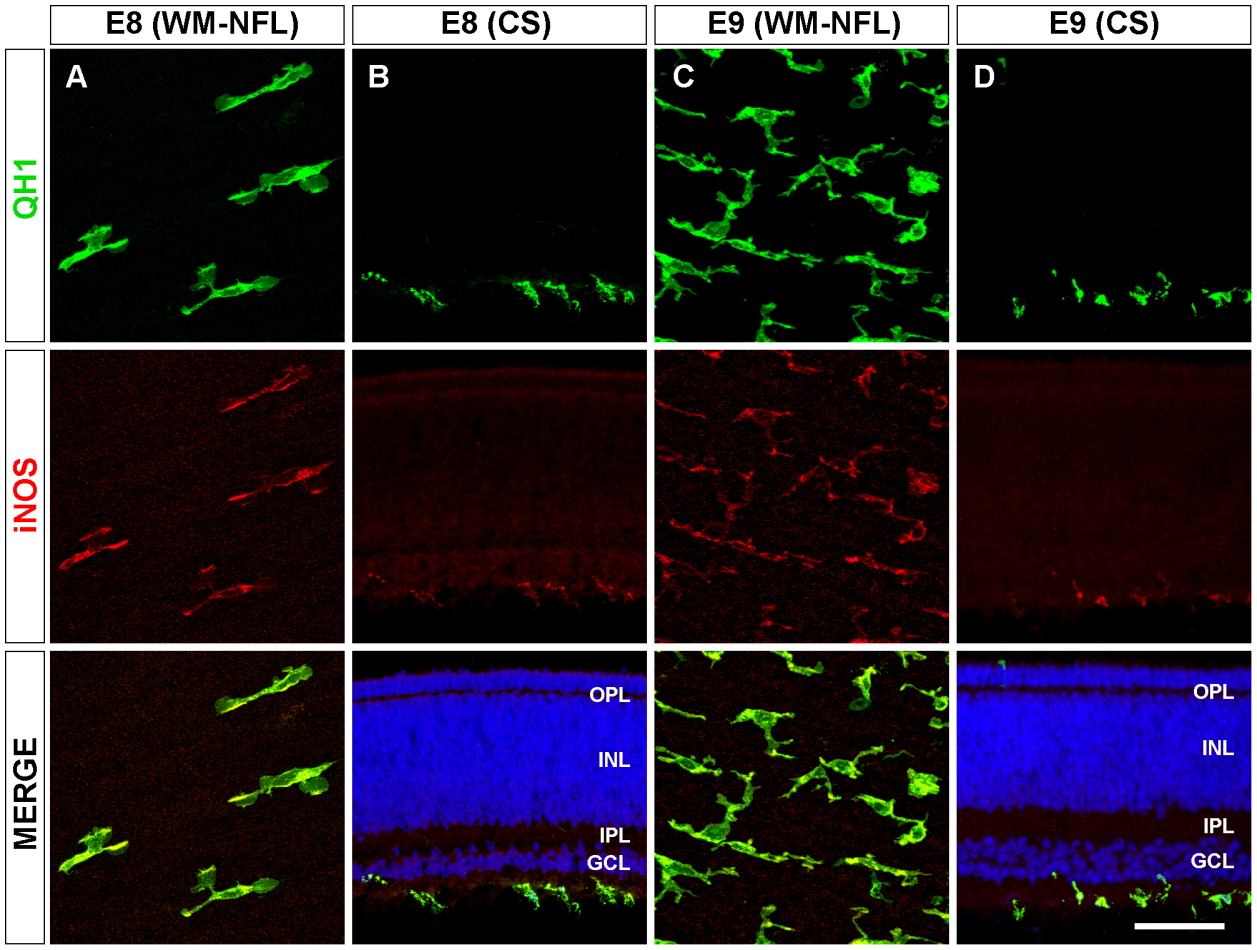
iNOS immunolabeling of amoeboid microglia in retinas of quail embryos at 8 and 9 days of incubation. Confocal micrographs of QH1 (green) and anti-iNOS (red) double-immunolabeled amoeboid microglial cells in whole-mounted retinas (WM) and retinal cross-sections (CS) of quail embryos at 8 (E8, **A**, **B**) and 9 days of incubation (E9, **C**, **D**). Cell nuclei in retinal cross sections are stained with Hoechst (blue). All QH1-positive amoeboid microglial cells in E8 and E9 embryo retinas are located in the nerve fiber layer (NFL) and are also immunolabeled with the anti-iNOS antibody. Anti-iNOS labeling of microglial cells is similar to QH1 labeling but with some differences. Thus, QH1 labels the entire microglial cell profile, including lamellipodia, whereas anti-iNOS labeling is exclusively cytoplasmic in distinct zones of the soma and cell processes. OPL: outer plexiform layer; INL: inner nuclear layer; IPL: inner plexiform layer; GCL: ganglion cell layer. Scale bar, 50 µm for A and C; 60 µm for B and D.

**Figure 2:**
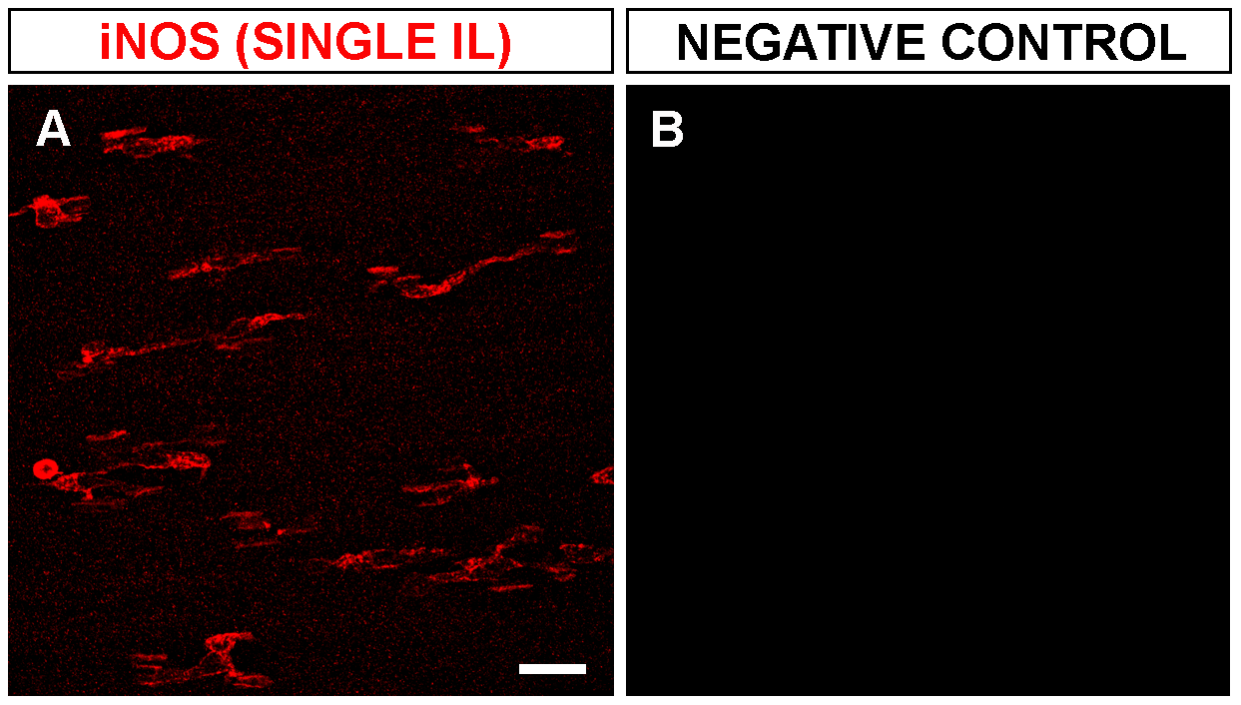
iNOS immunolabeling controls. Representative confocal images of single immunolabeling (IL) for iNOS (**A**) and its negative control, omitting the primary antibody (**B**) from whole-mounted retinas of quail embryos at 8 days of incubation. Note that the single iNOS labeling is similar to the iNOS labeling in double immunolabeled specimens shown in figure 1A, whereas no labeling is seen when anti-iNOS was omitted. Scale bar, 25 µm.

Previous studies by our group showed that amoeboid microglial cells enter the retina of quail embryos from the pecten/optic nerve head area between E7 and hatching (E16) and migrate tangentially in a central-to-peripheral direction on the vitreal part of the embryonic retina [60]–[62]. In the present study, immunofluorescence analysis in E8 and E9 quail embryo retinas showed that cells immunolabeled with the monoclonal antibody QH1, which recognizes microglial cells, were simultaneously immunostained with the polyclonal anti-iNOS antibody, whereas retinal neurons and Müller cells were not immunostained (Figure 1). No difference in results was observed between the use of two different polyclonal anti-iNOS antibodies (see Material and Methods), and cell labeling for iNOS was identical with single anti-iNOS immunofluorescence (Figure 2A) and with double QH1/iNOS immunostaining. Negative controls omitting the primary antibody showed no labeled cells (Figure 2B). Therefore, iNOS immunolabeling was specific to microglial cells.

**Figure 3:**
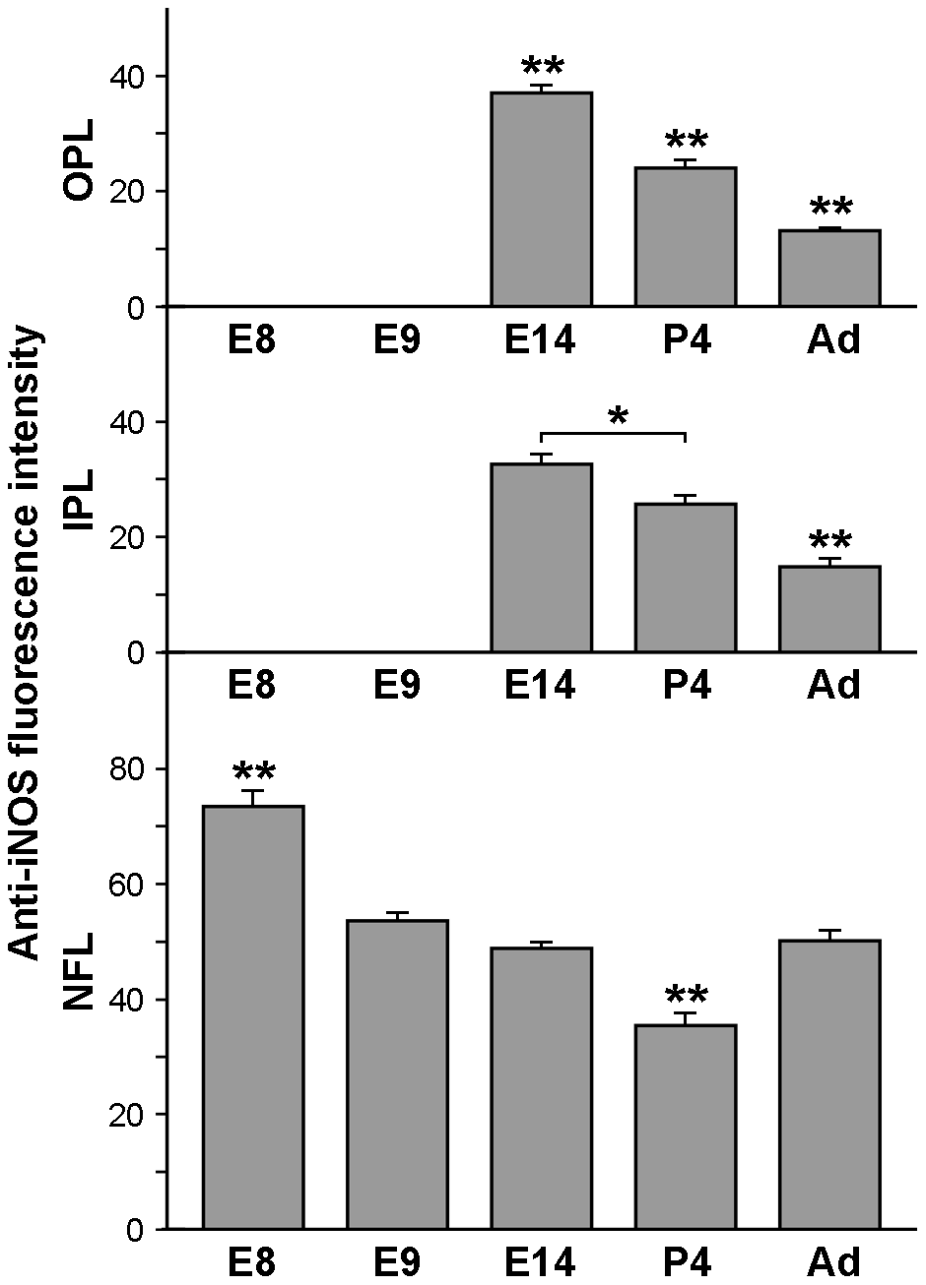
Anti-iNOS immunofluorescence intensity in microglial cells of quail embryo retinas from 8 days of incubation to adulthood. The histogram represents anti-iNOS fluorescence intensities per microglial cell obtained in the nerve fiber layer (NFL), inner plexiform layer (IPL) and outer plexiform layer (OPL) on confocal micrographs of double QH1/anti-iNOS immunolabeled whole-mounted retinas of quail embryos at 8 days of incubation (E8), E9, E14, and of 4-day-old (P4) and adult quails. Pixel intensities ranged from 0 (the darkest shade) to 255 (the lightest shade). Data are expressed as means ± SEM (n  =  30 cells for each age and retinal layer). Asterisks indicate significant differences (* p<0.05 and ** p<0.001, one-way ANOVA followed by Tukey test for multiple comparisons). Note that iNOS immunofluorescence intensities of microglial cells in the NFL are higher than in the IPL and OPL and show the highest value at E8, decreasing until P4 and then increasing in adulthood. In the IPL and OPL, the highest fluorescence intensity is observed at E14, with a significant decrease until adulthood. No data are shown for iNOS-immunofluorescence intensity in the IPL and OPL at E8 and E9 because no microglial cells were present in these layers at these ages.

**Figure 4:**
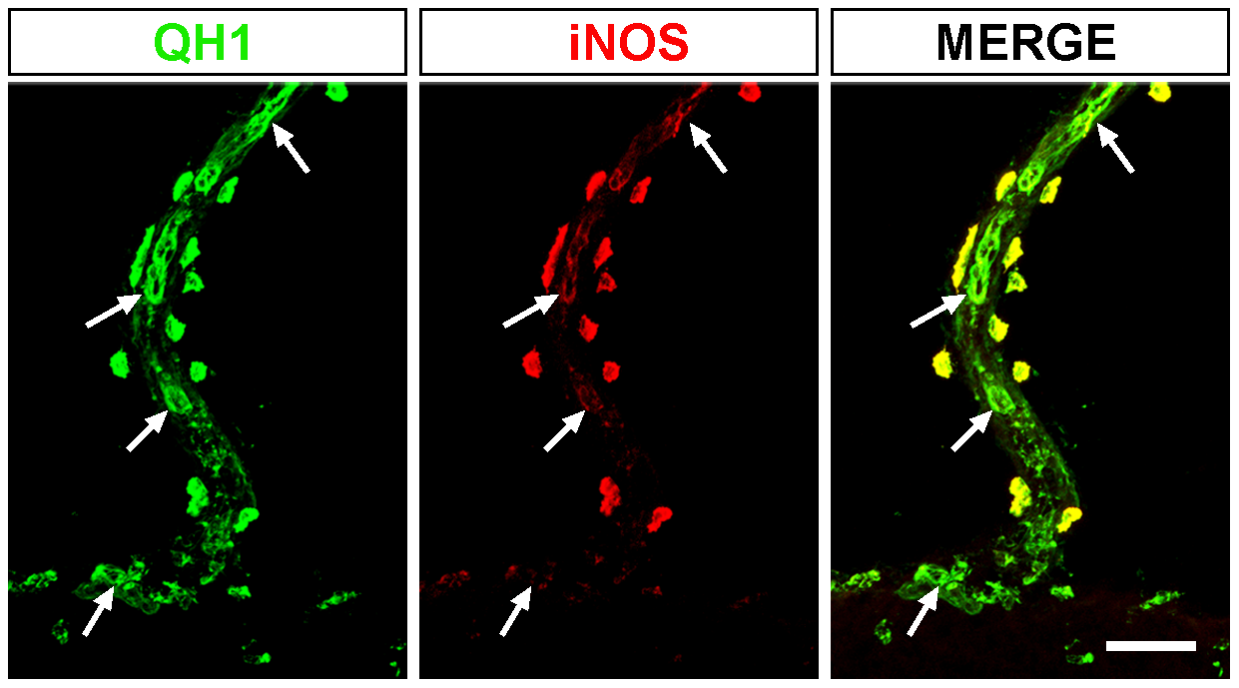
Pecten-adhered vitreal macrophages are also positive for iNOS immunostaining. Confocal image of a QH1 (green) and anti-iNOS (red) double-immunostained cross section of the pecten from a quail embryo at 8 days of incubation. Note that QH1-positive vitreal macrophages adhered to the pecten surface are strongly iNOS-positive, whereas endothelial cells of blood vessels (arrows) within the pecten show strong QH1 labeling but are weakly immunostained with anti-iNOS. Scale bar, 25 µm.

Microglial cells in E8 retinas were exclusively amoeboid and localized in the NFL, where they were migrating tangentially from the center to the periphery [60], [62]. These amoeboid microglial cells were strongly labeled with anti-iNOS in both whole-mounted retinas (Figure 1A) and retinal cross sections (Figure 1B). QH1/iNOS double immunolabeling of E9 retinas also revealed iNOS-positive amoeboid microglial cells in the vitreal part of the retina (Figure 1C, D), although they were more abundant than in E8 retinas and showed a lower anti-iNOS fluorescence intensity (Figures 1 and 3).The iNOS immunolabeling of amoeboid microglial cells bore some resemblance to the QH1 immunostaining, but they were clearly different (compare QH1 and iNOS micrographs in Figure 1A, C). Thus, QH1 labeling showed the entire microglial cell profile, including lamellipodia (Figure 1A, QH1 panel), because this antibody labels both the cytoplasmic structures and the cell membrane of microglial cells [61]. However, anti-iNOS labeling was exclusively cytoplasmic in discrete zones of the soma and some cell processes but lamellipodia were not immunostained (Figure 1A, iNOS panel). iNOS-positive microglial cytoplasmic components were always QH1-positive. In addition, QH1-positive vitreal macrophages adhering to the pecten surface were strongly iNOS-positive, whereas endothelial cells of the pecten blood vessels were strongly immunolabeled with QH1 but very weakly immunostained with anti-iNOS (Figure 4), a further difference between the iNOS and QH1 immunolabeling.

### Decreased iNOS immunolabeling in ramified microglial cells of the developing and adult quail retina

**Figure 5:**
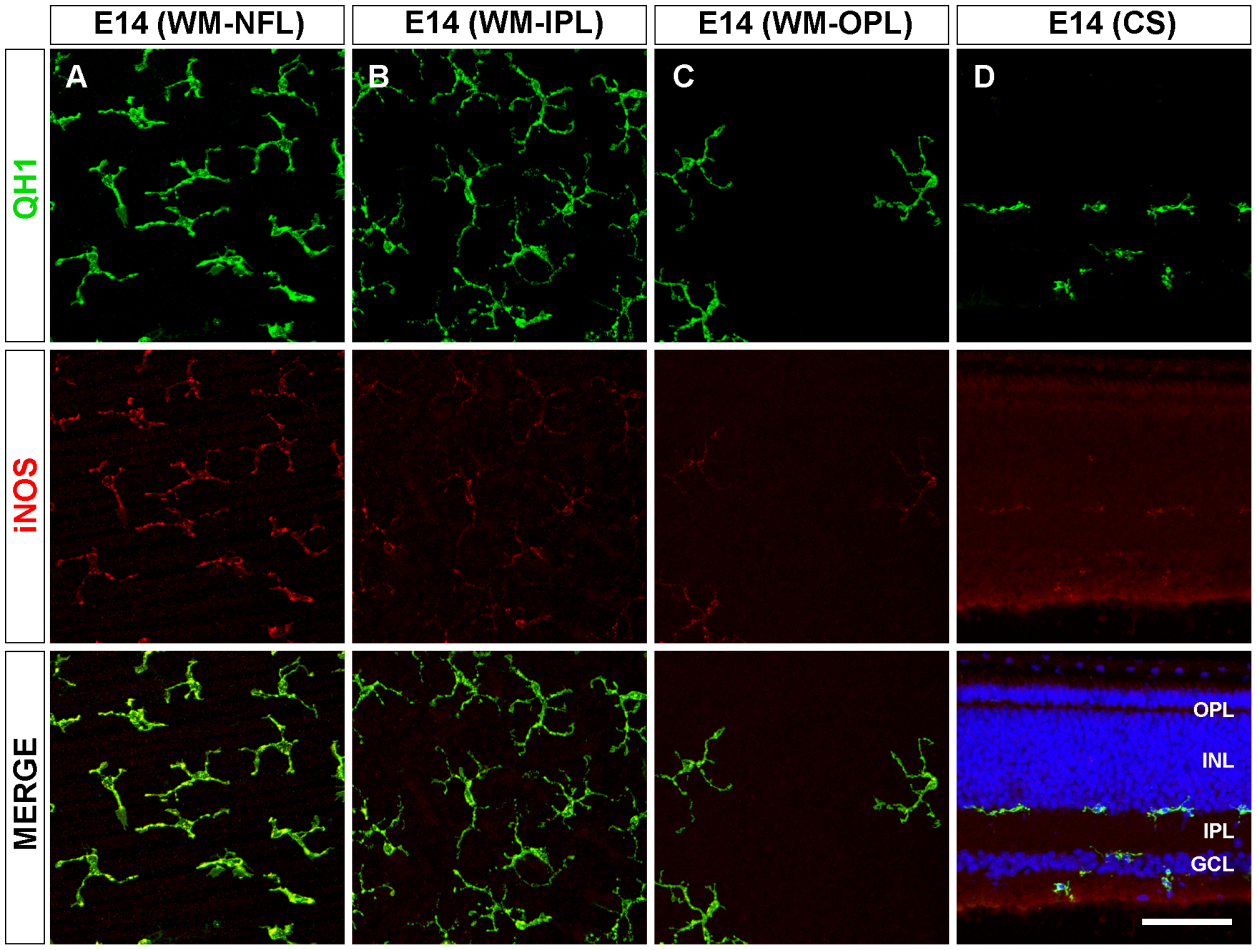
Decreased iNOS immunolabeling in ramifying microglial cells at the end of embryonic development of the quail retina. Representative confocal images of QH1 (green) and anti-iNOS (red) double-immunolabeled microglial cells in a whole-mounted retina (WM, **A-C**) and a retinal cross section (CS, **D**) of quail embryos at 14 days of incubation (E14). Cell nuclei in the retinal cross section are stained with Hoechst (blue). In the nerve fiber layer (NFL), microglial cells have an amoeboid appearance with strong iNOS immunolabeling (**A, D**). However, in the inner plexiform layer (IPL) and outer plexiform layer (OPL), ramifying microglial cells have a weaker iNOS labeling (**B-D**), suggesting that iNOS expression becomes downregulated as amoeboid microglia differentiate into ramified microglia. INL: inner nuclear layer; GCL: ganglion cell layer. Scale bar, 50 µm for A-C; 60 µm for D.

Between E9 and P3, microglial cells migrate radially in a vitreal-to-scleral direction to reach their final location in the IPL and OPL, where they ramify [60], [62], [63]. Thus, in E14 quail embryo retinas, many microglial cells had already reached the IPL and OPL (Figure 5). In the NFL, microglial cells continued to have an amoeboid appearance, with morphological features similar to those seen at E8 and E9, and they showed strong iNOS immunolabeling (Figures 3 and 5A). In the IPL, abundant ramifying microglial cells were observed, with a weaker iNOS labeling in comparison to the amoeboid microglia in the NFL (Figures 3 and 5B), suggesting that iNOS expression was downregulated as amoeboid cells differentiated into ramified microglia. Similar observations were made in the OPL, although microglial cells were scarce in this layer and were less ramified than in the IPL (Figures 3 and 5C).

**Figure 6:**
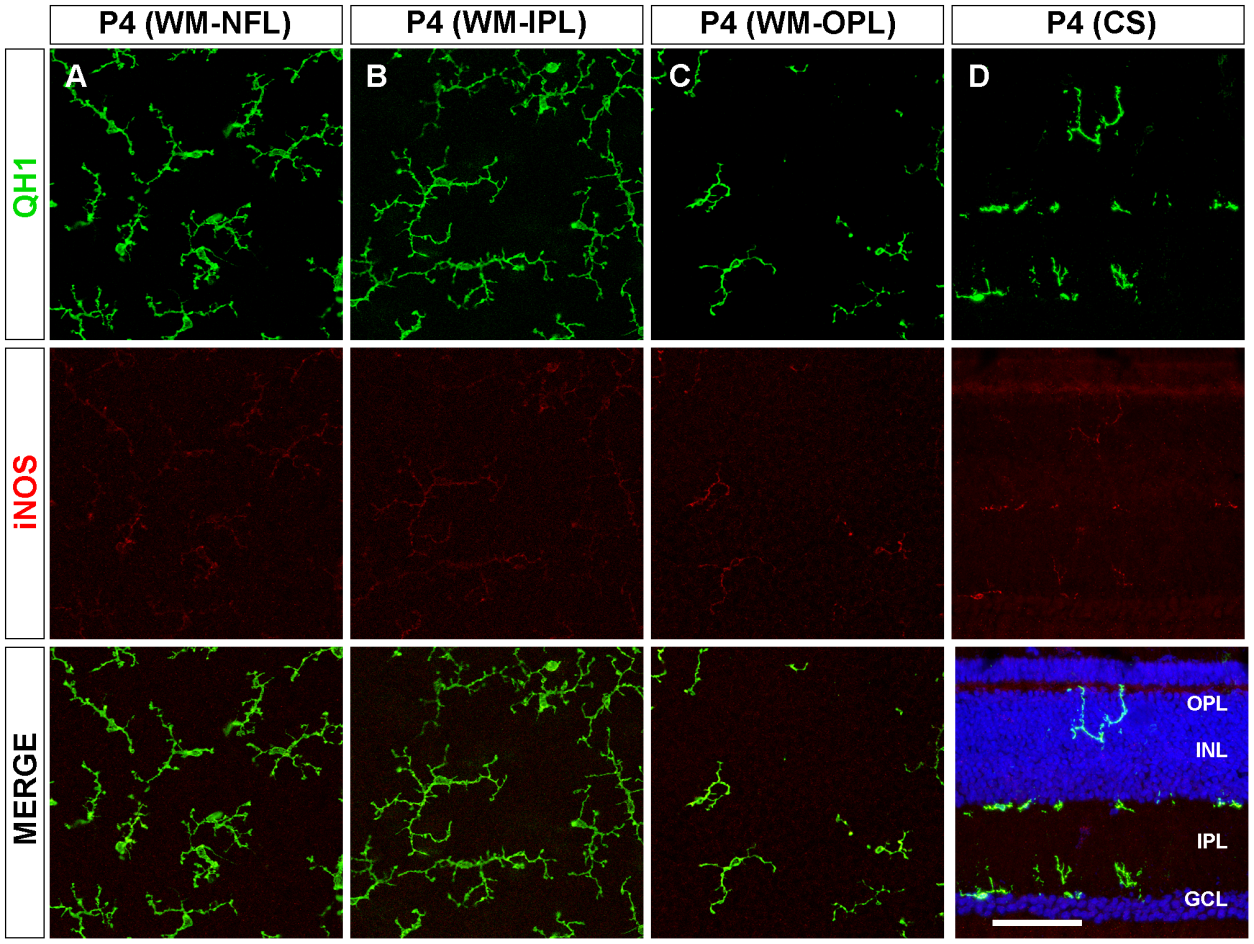
Weak iNOS immunolabeling of ramified microglia in the developing quail retina during the first post-hatching week. Representative confocal images of QH1 (green) and anti-iNOS (red) double-immunolabeled microglial cells in a whole-mounted retina (WM, **A-C**) and retinal cross sections (CS, **D**) from 4-day-old quails (P4). Hoechst staining was used to visualize nuclei (blue) in the retinal cross section. Microglial cells at this developmental stage are mainly located in the nerve fiber layer (NFL, **A**), inner plexiform layer (IPL, **B**), and outer plexiform layer (OPL, **C**) and show a more profuse ramification and a similar anti-iNOS immunostaining in comparison to ramifying microglial cells in retinas from embryos at 14 days of incubation. During the first post-hatching week, some microglial cells can be seen in the inner nuclear layer (INL), apparently traversing this layer towards the OPL (**D**). GCL: ganglion cell layer. Scale bar, 50 µm for A-C; 65 µm for D.

Microglial cells in the NFL, IPL, and OPL of P4 retinas were more profusely ramified than at E14 (Figure 6). Interestingly, iNOS immunostaining of ramified microglia was significantly lower in the NFL, IPL and OPL of P4 retinas than in the same layers at E14 (Figures 3 and 6).

**Figure 7:**
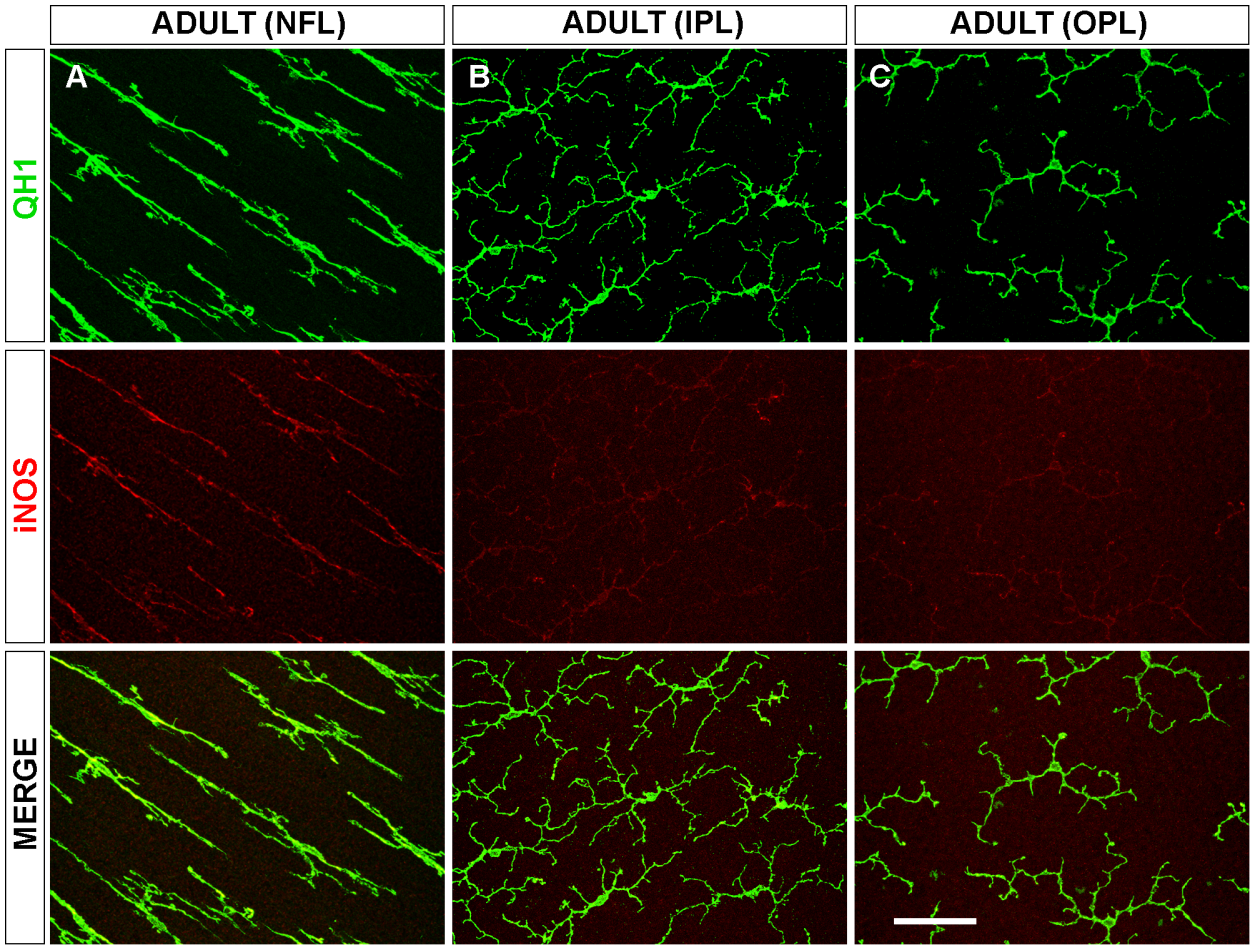
iNOS immunolabeling of ramified microglial cells in the adult quail retina is weak in the plexiform layers and stronger in the nerve fiber layer. Representative confocal images of a QH1 (green) and anti-iNOS (red) double-immunolabeled adult whole-mounted retina showing mature ramified microglial cells in the nerve fiber layer (NFL, **A**), inner plexiform layer (IPL, **B**), and outer plexiform layer (OPL, **C**). In the NFL, microglial cell processes are parallel to the course of ganglion cell axon fascicles (not shown) and show stronger iNOS immunolabeling in comparison to ramified microglial cells in the IPL and OPL, whose cell processes are oriented in all directions. Microglial iNOS immunostaining in the IPL and OPL is similar to that in retinas from quail embryos at 14 days of incubation and from 4-day-old quails. Scale bar, 50 µm.

In the adult retina, microglial cells had achieved their mature ramification pattern in the NFL, IPL, and OPL [64] (Figure 7). Cell processes of ramified microglia were parallel to the course of ganglion cell axon fascicles in the NFL (Figure 7A, QH1 panel) but were oriented in all directions in the IPL (Figure 7B, QH1 panel) and OPL (Figure 7C, QH1 panel), where microglial cells had a star-like appearance. The iNOS immunolabeling of mature ramified microglia was significantly lower in the IPL and OPL of adult retinas than in these layers at P4 (Figures 3 and 7B, C, iNOS panels). However, iNOS immunostaining of the elongated microglial cells in the NFL was stronger at adulthood than at P4 (Figures 3 and 7A, iNOS panel).

### iNOS mRNA expression throughout quail retina development

**Figure 8:**
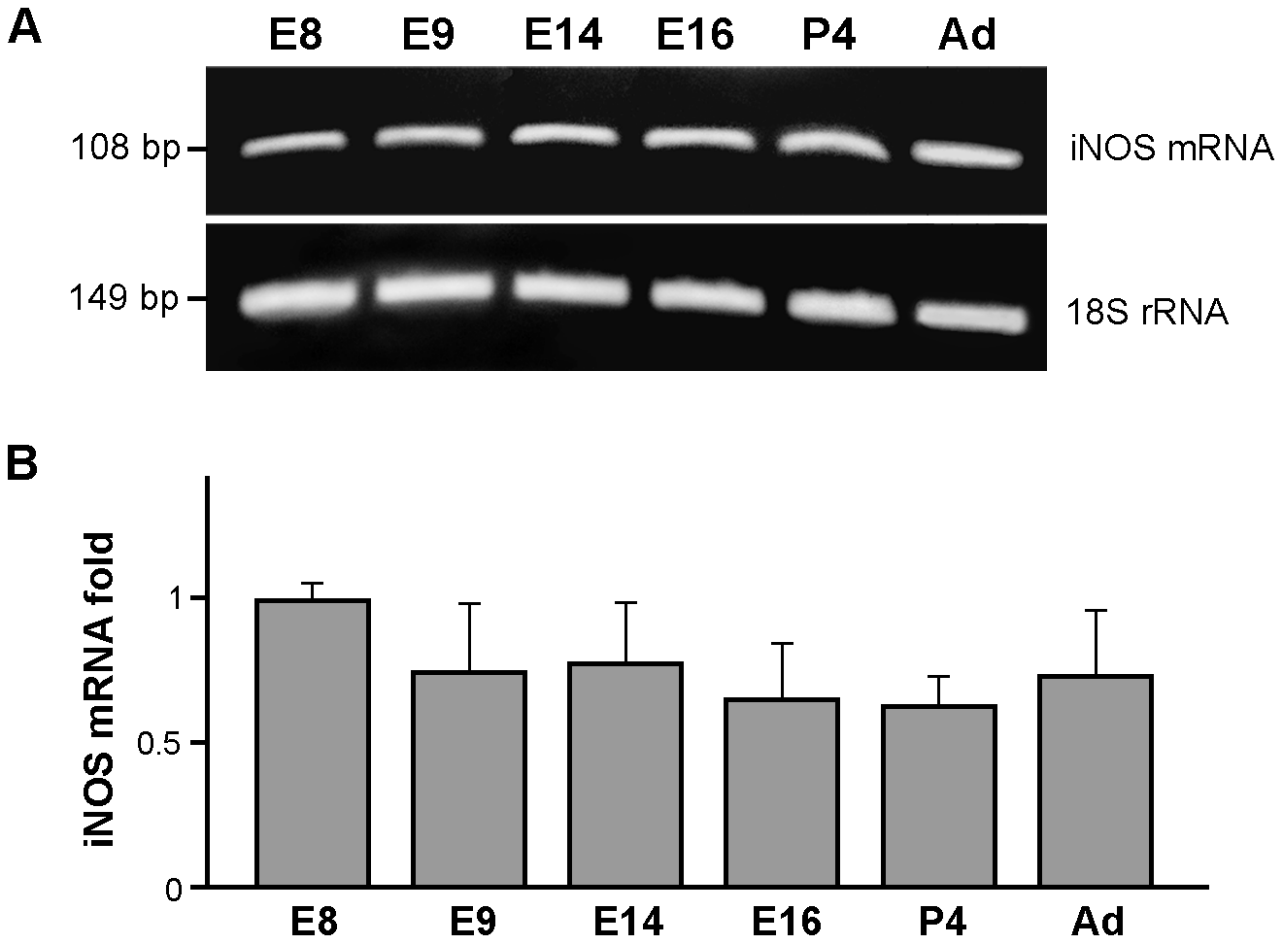
iNOS gene is expressed in the quail retina during embryonic and post-hatching development and adulthood. **A**. Representative gel from three independent experiments of agarose gel electrophoresis of iNOS mRNA by RT-PCR of cDNAs prepared from quail retinas of different embryonic (E8, E9, E14 and E16) and post-hatching (P4 and adulthood) ages. iNOS RT-PCR products at each age are referred to the corresponding 18S rRNA bands. Expression of the iNOS gene is observed in retinas at all tested ages (embryonic, postnatal and adulthood). **B**. Quantitative analysis of iNOS mRNA expression in quail retinas of different ages, as shown by RT-PCR of six RT-PCR experiments. The histogram represents changes in the iNOS mRNA levels (mean ± SEM) at the different ages with respect to E8. No significant differences are observed between the different ages (one-way ANOVA followed by Tukey test for multiple comparisons).

RT-PCR analysis of iNOS mRNA from E8, E9, E14, E16, P4 and adult retinas demonstrated iNOS gene expression in the quail retina during embryonic and postnatal development and in adulthood (Figure 8A). We highlight that the iNOS mRNA level in E8 retinas, as quantified by RT-PCR analysis, did not significantly differ from that in developing retinas from E9 onward and in adult retinas (Figure 8B), contrasting with the reduction in iNOS immunolabeling from E9 onward (Figure 3). These apparently contradictory results can be explained by the presence of relatively few microglial cells in E8 retinas and the subsequent increase in their number with higher age [60]. Given that the amount of total RNA used to synthesize cDNA and amplify iNOS gene cDNA was always the same, the higher concentration of iNOS mRNA per cell from a low number of E8 retina microglial cells would match the lower amount of mRNA per cell from a higher number of microglial cells in more developed retinas.

### Increased activation of amoeboid microglia after *in vitro* LPS treatment of explants from E8 quail embryo retina

Strong iNOS immunolabeling of amoeboid microglia in E8 quail embryo retinas suggest some degree of activation, as described in the CNS of developing mice [40], [41]. In order to test whether amoeboid microglia were able to increase their activation degree in response to toxic exogenous factors, experiments were made on organotypic cultures of E8+24hiv retina explants incubated in the presence of LPS.

**Figure 9:**
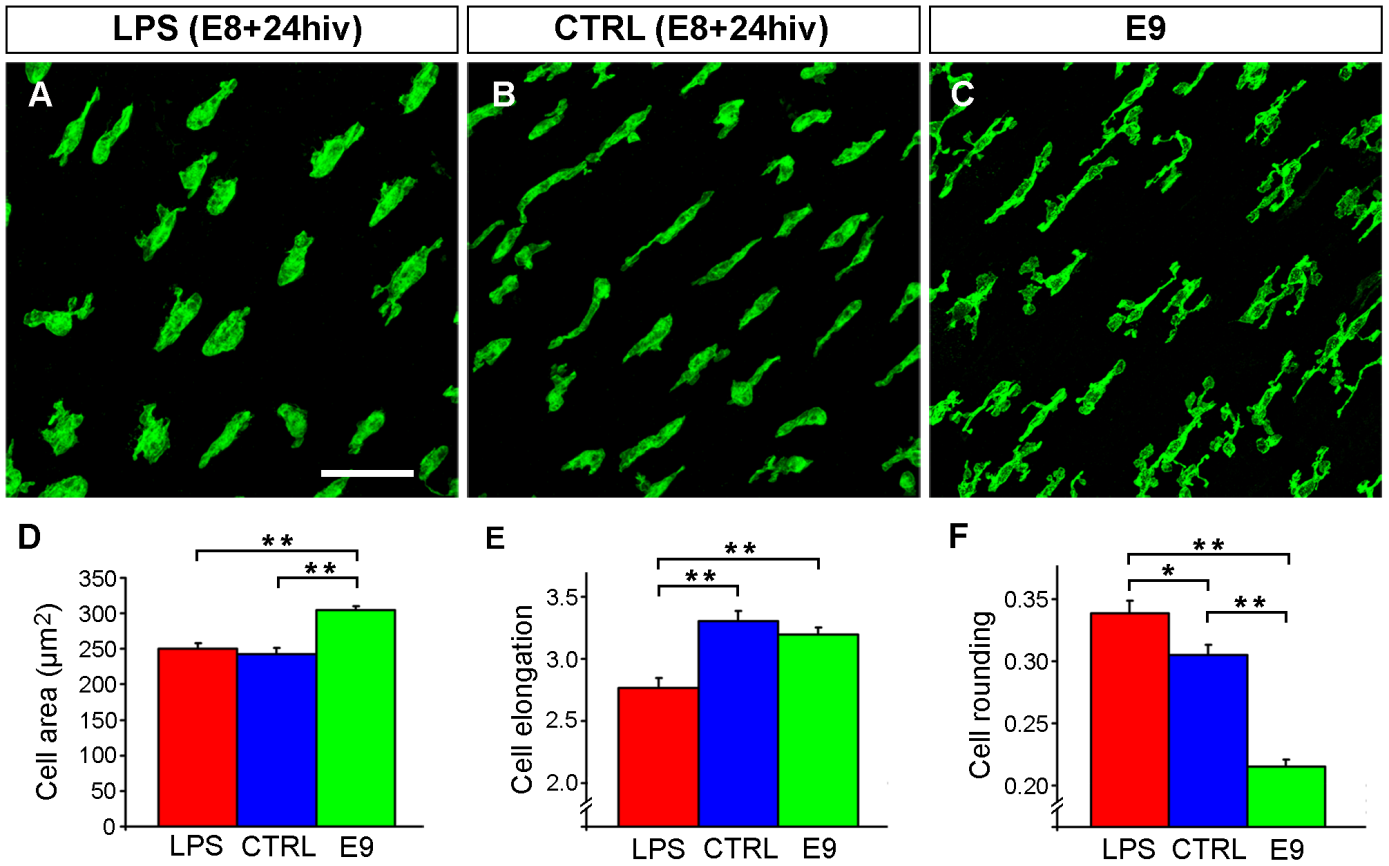
In vitro LPS treatment of quail embryo retina explants induces morphological changes in amoeboid microglia compatible with activation. **A, B**. Representative images of a pair of QH1 immunolabeled retina explants from a quail embryo at 8 days of incubation cultured for 24 hrs *in vitro* (E8+24hiv) in medium containing LPS (LPS, **A**) and in LPS-free medium (CTRL, **B**). QH1-positive microglial cells in the LPS-treated explant are more rounded than those in the control explant. **C**. QH1 immunolabeled non-cultured retina explant from a quail embryo at 9 days of incubation (E9), showing the typical polarized morphology of amoeboid microglia migrating tangentially in the nerve fiber layer, with elongated cell body, polarized cell processes, and broad lamellipodia. Note that the microglial cell morphology is similar in the control E8+24hiv retina explant (**B**) to that in the non-cultured E9 retina explant (**C**), although lamellipodia are noticeably less abundant in the former. **D-F**. Morphometric analysis of cell area (**D**), elongation index (**E**), and cell rounding index (**F**) for microglial cells in LPS-treated E8+24hiv (red bars), non-treated control E8+24hiv (blue bars), and non-cultured E9 (green bars) retina explants. Data are expressed as means ± SEM (n = 30 for each). Asterisks indicate significant differences (* p<0.05 and ** p<0.001, one-way ANOVA followed by Tukey test for multiple comparisons). Scale bar, 50 µm.

Microglial cells in non-cultured E8 retina explants were exclusively located in the NFL and showed a polarized morphological appearance typical of tangentially-migrating amoeboid microglia (Fig. 1A), with an elongated cell body bearing two cell processes in opposite poles and broad lamellipodia, as previously described [54]. After LPS treatment for 24 hiv, microglia in E8 retina explants changed their morphological features, showing a more rounded cell body with scarce, shorter cell processes and infrequent lamellipodia (Figure 9A). Control E8+24hiv explants were cultured in medium without LPS and showed that microglial cells retained an elongated morphology (Figure 9B) similar to that of microglia in non-cultured E8 retina explants. Non-cultured explants from E9 retinas were also used to compare the morphological appearance of microglia in control E8+24hiv explants (Figure 9B) with that in the non-cultured retina at an equivalent developmental age (Figure 9C). Morphological polarization of microglial cells in control E8+24hiv explants was similar to that in non-cultured E9 retina explants, although lamellipodia were noticeably less abundant in the former (compare B and C in Figure 9).

Morphometric analysis revealed that the microglial cell area was similar between LPS-treated and control E8+24hiv explants but significantly higher in E9 retinas (Figure 9D). These differences in cell area appear attributable to the presence of abundant broad lamellipodia in microglial cells of non-cultured E9 retinas, which were retracted during *in vitro* culture of E8 explants. The cell elongation index was significantly lower in LPS-treated E8+24hiv explants than in control E8+24hiv explants or E9 retinas (Figure 9E), whereas the cell rounding index was significantly higher in the former (Figure 9F). Accordingly, LPS treatment of retina explants induced important changes in the phenotype of microglial cells compatible with increased activation.

**Figure 10:**
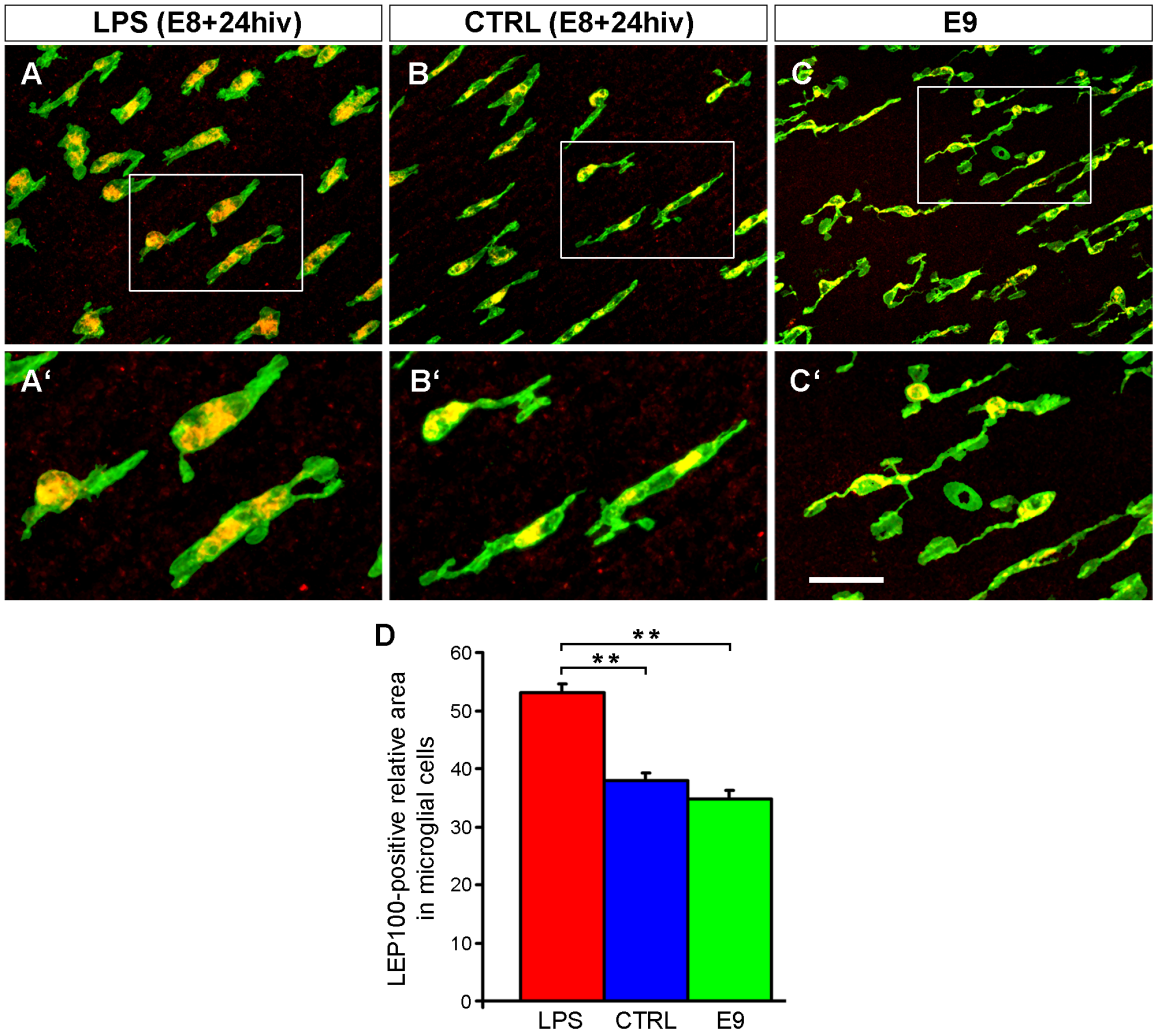
LPS treatment markedly increases the lysosomal compartment in amoeboid microglia of quail embryo retina explants. Confocal images of QH1 (green) and LEP100 (specific lysosomal membrane marker, red) double-immunolabeled amoeboid microglial cells in retina explants from quail embryos at 8 days of incubation cultured for 24 hrs *in vitro* (E8+24hiv) in medium containing LPS (LPS, **A**, **A′**) and in LPS-free medium (CTRL, **B**, **B′**). Double-immunolabeled microglial cells in non-cultured retina explants from quail embryos at 9 days of incubation (E9) are shown in **C** and **C′**. The lysosomal compartment is clearly larger in the LPS-treated E8+24hiv retina explant than in control E8+24hiv and non-cultured E9 retina explants. **D**. Morphometric analysis shows that the relative area of the microglial cell lysosomal compartment is significantly higher in LPS-treated E8+24hiv retina explants (red bar) than in non-treated control E8+24hiv (blue bar) or non-cultured E9 (green bar) retina explants. Data are expressed as means ± SEM (n = 45 for each). Asterisks indicate significant differences (* p<0.05 and ** p<0.001, one-way ANOVA followed by Tukey test for multiple comparisons). Scale bar, 50 µm for A-C; 23 µm for A′-C′.

Given that the activation process induces an increase in lysosomal protein synthesis [65], microglial activation after LPS treatment of E8 retina explants was also investigated by analyzing the lysosomal compartment revealed by LEP100 antibody immunolabeling (Figure 10). This compartment was mainly located in the cell body of microglial cells and was clearly larger in LPS-treated E8+24hiv explants (Figure 10A, A′) than in control E8+24hiv explants (Figure 10B, B′) or E9 retinas (Figure 10C, C′). Morphometric analysis confirmed these observations, showing that the relative area occupied by the lysosomal compartment with respect to the total microglial cell area was significantly higher (around 15%) in LPS-treated E8+24hiv explants than in the control or E9 retinas (Figure 10D). According to these results, amoeboid microglia in E8 retina explants increased their activation after LPS treatment for 24 hiv.

A study was also conducted of variations in the morphology of microglial cells after LPS treatment of retinal explants for 24 hiv. The typical rounded microglial phenotype of activated cells was already observed at 1 hiv, indicating that microglial activation was induced by LPS soon after its addition to the culture medium and was subsequently maintained (results not shown).

### Increased iNOS expression in activated amoeboid microglia of LPS-treated retina explants

**Figure 11:**
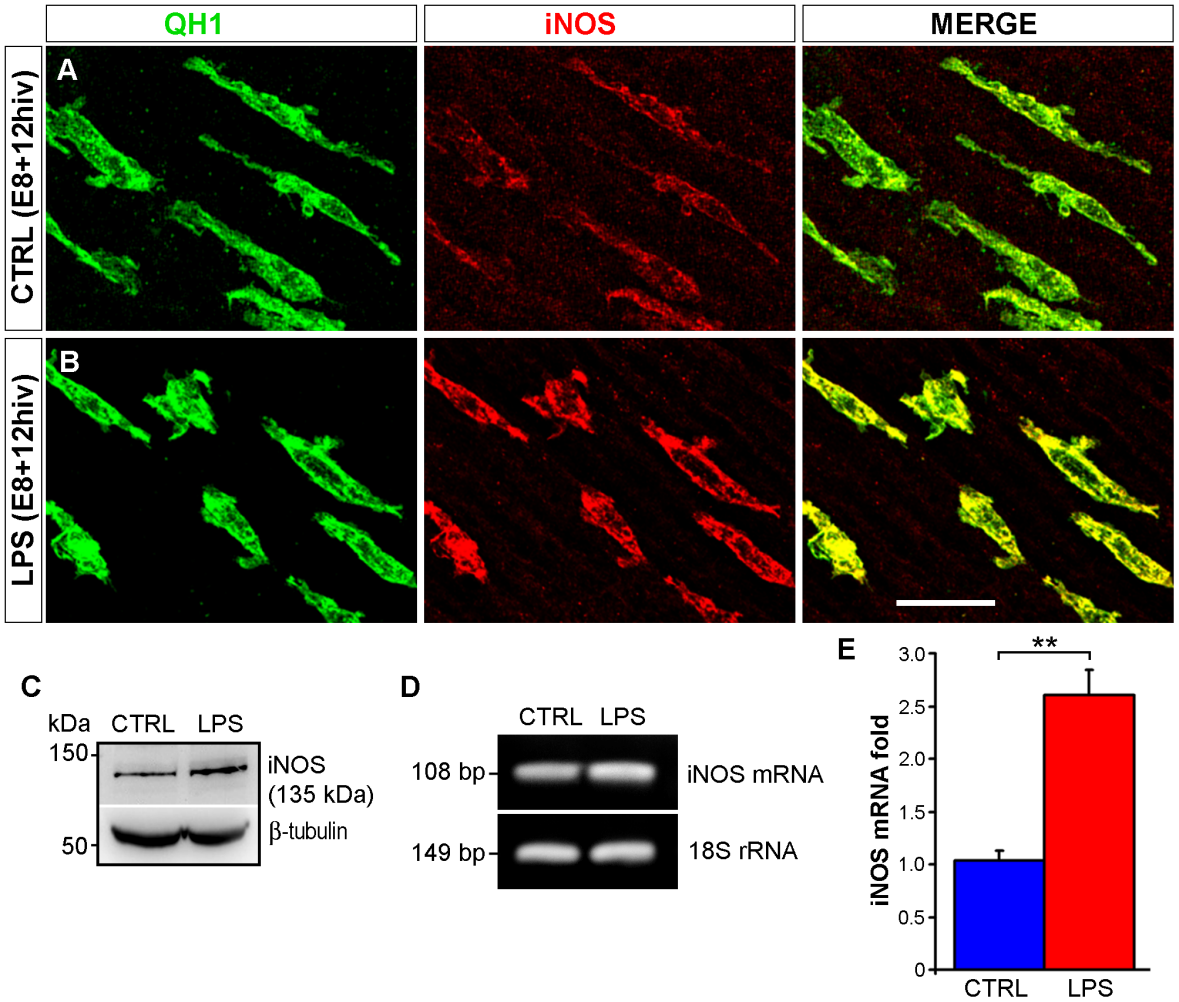
iNOS gene expression is upregulated in LPS-treated quail embryo retina explants cultured for 12 hrs *in vitro* (hiv). **A**, **B**. Confocal images of QH1 (green) and anti-iNOS (red) double immunostained microglial cells in non-treated control (CTRL, **A**) and LPS-treated (LPS, **B**) retina explants from quail embryos at 8 days of incubation cultured for 12 hiv (E8+12hiv). The iNOS labeling is higher in LPS-treated *versus* control explants. **C**. Western blot analysis results, representative of three independent experiments, for iNOS protein levels in non-treated control (CTRL) and LPS-treated (LPS) E8+12hiv retina explants. The 135 kDa band, which corresponds to the iNOS protein, is noticeably more intense in LPS-treated *versus* control explants. β-tubulin antibody was used as a loading control. **D**. Representative gel of three independent experiments on agarose gel electrophoresis of iNOS mRNA, showing a more intense band in LPS-treated explants (LPS) than in control explants (CTRL). iNOS amplification products are referred to the corresponding 18S rRNA bands. **E**. Quantitative analysis of iNOS mRNA expression by real-time PCR. The histogram represents changes in the iNOS mRNA levels of LPS-treated E8+12hiv retina explants with respect to non-treated explants (mean ± SEM) obtained from three real-time PCR experiments. iNOS mRNA levels are more than two-fold higher in LPS-treated explants than non-treated explants. Scale bar, 25 µm.

A major difference in the iNOS labeling of microglia between control and LPS-treated E8 explants was observed at 12 hiv (compare A with B in Figure 11). Therefore, this culture time was chosen for western blot analysis of possible differences in iNOS protein levels between non-treated and LPS-treated explants. Immunoblots of retina explant lysates showed that a 135-kD band, corresponding to the iNOS protein, was noticeably more intense in LPS-treated *versus* control explants (Figure 11C). Determination of iNOS mRNA expression in E8 explants by RT-PCR showed that iNOS mRNA levels were more than two-fold higher in LPS-treated *versus* non-treated explants (Figure 11D, E), reflecting an upregulation of the iNOS gene after LPS treatment.

**Figure 12:**
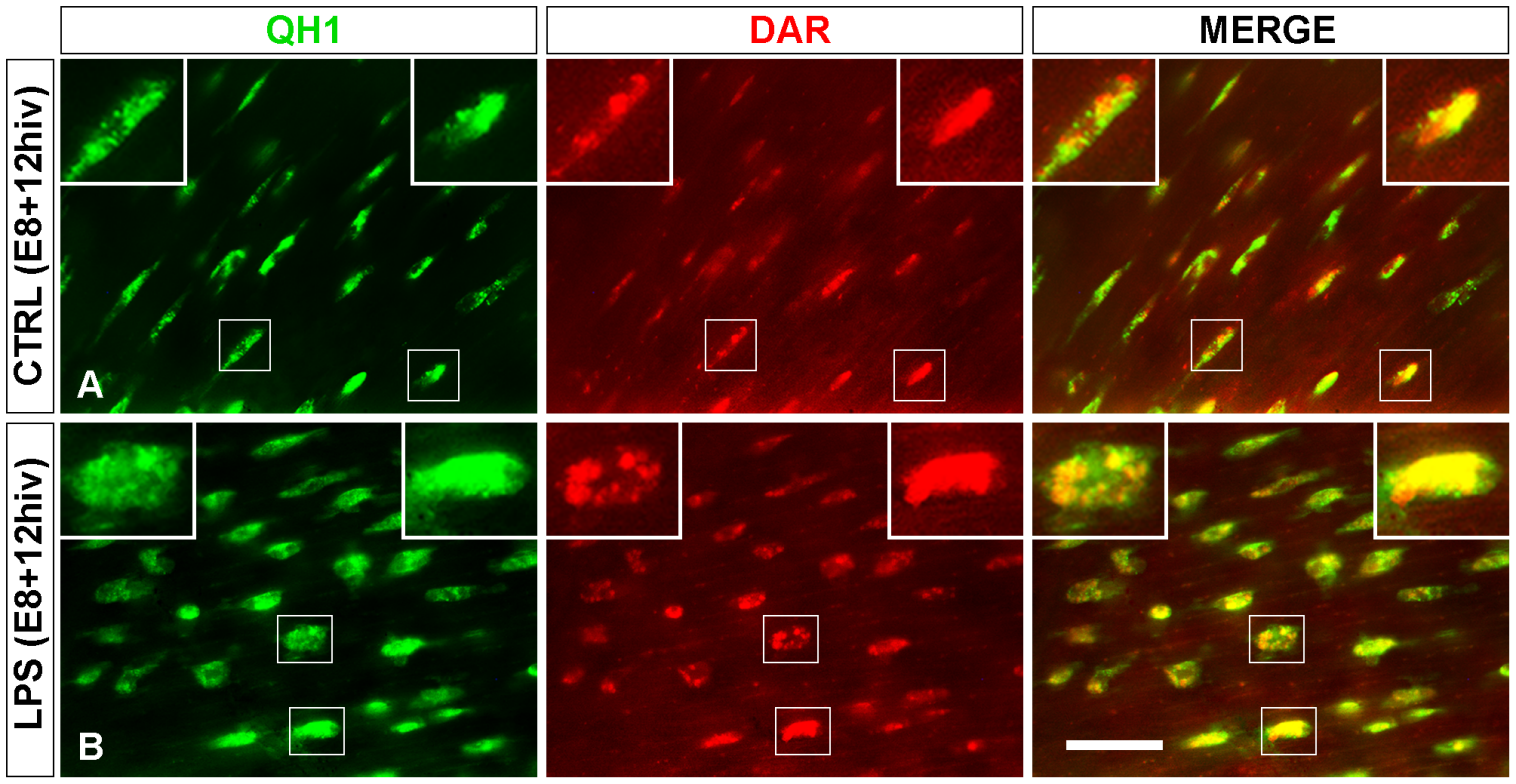
Microglial cells produce NO in quail embryo retina explants from quail embryos at 8 days of incubation cultured for 12 hrs *in vitro* (E8+12hiv). Representative images of QH1 (green) immunolabeled amoeboid microglia in non-treated control (CTRL, **A**) and LPS-treated (LPS, **B**) E8+12hiv retina explants cultured in the presence of DAR-4M AM (DAR, red), a reliable fluorescent indicator of subcellular NO production by iNOS. All microglial cells are specifically labeled with DAR in the LPS-treated and control explants. An entirely DAR-fluorescent cell body is observed in some cells (right inserts), whereas other cell bodies contain several distinct DAR-fluorescent masses of variable size (left inserts). Scale bar, 50 µm; 20 µm for inserts.

DAR-4M AM, a reliable fluorescent indicator of subcellular NO production by iNOS [58], [59], was used to determine the true production of NO in amoeboid microglial cells of control and LPS-treated E8+12hiv retina explants, which were previously shown to express iNOS. DAR-4M AM fluorescence was detected in elongated amoeboid microglial cells of non-treated control E8+12hiv explants (Figure 12A), reflecting NO production in these cells, and was also detected in activated rounded microglial cells of LPS-treated E8+12hiv explants (Figure 12B). DAR-4M AM fluorescence was not detected in non-QH1 labeled cells in either control or LPS-treated explants, verifying that NO production was specific to microglial cells. The morphology of DAR-4M AM fluorescence was variable. Thus, a DAR-4M AM-fluorescent mass filling the entire cell body was observed in some cells (right inserts in Figure 12A, B), whereas others had cell bodies containing more or less rounded DAR-4M AM-fluorescent masses of different sizes (left inserts in Figure 12A, B). At any rate, the appearance of DAR-4M AM fluorescence in each microglial cell differed from that of the QH1-labeling.

Taken together, our results demonstrate that LPS treatment of E8 retina explants induced a higher activation degree in amoeboid microglia, which resulted in significant iNOS upregulation.

## Discussion

The results of this study demonstrate that amoeboid microglia express iNOS and produce NO during normal embryonic development of the quail retina. Given the association between iNOS expression and microglial activation [13], [34], [37], these findings indicate a certain degree of activation (“baseline activation”) in amoeboid microglia during normal development. iNOS expression becomes downregulated as amoeboid microglia differentiate into ramified microglia, suggesting a decrease in this baseline activation during the microglial differentiation process. We also found that LPS treatment of organotypic cultures of E8 retina explants *in vitro* induces amoeboid microglia to change their morphology, increase their lysosomal compartment, and upregulate their iNOS expression, with a concomitant increase in NO production. We conclude from these results that amoeboid microglia in the quail embryo retina increase their activation level in response to the action of LPS.

### iNOS expression and NO production by normal amoeboid microglia

Non-pathological amoeboid microglia migrating in the NFL of E8 and E9 quail embryo retina were shown to express iNOS and produce NO. These observations were made after specifically immunolabeling amoeboid microglia with polyclonal antibodies against mouse iNOS, which are described by the manufacturers as showing reactivity with chick iNOS. It could be argued that these anti-iNOS polyclonal antibodies might have cross-reacted with an antigen distinct from iNOS, given the heterogeneity of polyclonal antibodies. However, iNOS expression in amoeboid microglia of developing quail retina was confirmed by the results of three other investigations. First, analysis of the iNOS mRNA by RT-PCR revealed iNOS expression in normal developing retinas, which presumably corresponds to microglial cells because the NOS expressed by neurons is generally nNOS [36]. Second, *in vitro* experiments using organotypic cultures of E8 quail embryo retina explants incubated for 24 hiv showed that amoeboid microglia upregulate iNOS expression in response to LPS treatment, as revealed by: their increased anti-iNOS immunolabeling, greater western blot-determined iNOS protein content, and higher RT-PCR-analyzed iNOS mRNA levels. Third, the specific microglial production of NO, the final molecule catalyzed by iNOS action, was revealed by fluorescent probe using DAR-4M AM. This fluorophore has proven to be a reliable indicator of NO production [58], [59] and has been employed in numerous studies [66]–[69]; therefore, the specific DAR-4M AM labeling of microglial cells can be considered conclusive evidence of iNOS expression in normal amoeboid microglia. In the present study, DAR-4M AM was used in E8+12hiv retina explants to avoid technical problems inherent to its *in vivo* utilization. Given the physiologic behavior of microglial cells in E8 quail retina explants up to 24 hiv [54], microglial NO production in these explants would be representative of the production *in vivo*. The upregulation of iNOS expression in response to LPS treatment provides further indirect evidence of iNOS expression in normal retina amoeboid microglia. This upregulation is consistent with the well-documented effect of LPS in other systems [19], [50], [52], [70] and was demonstrated in our study by anti-iNOS immunocytochemistry, western blot, quantitative RT-PCR, and DAR-4M AM-based NO detection, validating the technical procedure used to reveal iNOS expression in microglial cells of *in vivo* retinas.

The finding of iNOS expression in amoeboid microglia during normal retina development conflicts with the widely held view that it is only expressed by microglia in pathological events as a response to inflammatory factors [17], [18], [34], [36], [52], although iNOS expression has also been reported in the normal adult human retina [71]. A previous study [72] showed that NOS activity was elevated in the retinas of E8-E9 chick embryo retinas and decreased thereafter. Various retinal neurons are known to express nNOS in developing and adult chicks [73]–[77], and eNOS has been detected in certain neuronal populations and in Müller cells [73], [78]. However, there has been no previous report of iNOS expression in any cell type of the normal retina of avian species. Our results show that iNOS is expressed in microglia of the non-pathological quail retina, mainly during development. Another study using NADPH-diaphorase histochemistry revealed NOS activity in amoeboid microglia during the postnatal development of rat corpus callosum and internal and external capsules [79]. This was attributed by the authors to iNOS, given the absence of nNOS immunolabeling in the microglia. Recently, iNOS expression was also reported in amoeboid microglia of prenatal rat neocortex [38] and 3-day-old mouse whole brain [39]. Nevertheless, a recent study showed that iNOS is only detected in rare microglial cells in the corpus callosum of postnatal transgenic mice expressing the fluorescent reporter tdTomato and CRE recombinase under the control of iNOS gene regulatory regions [52]. Constitutive iNOS expression has also been described in other non-pathological brain cell types, such as early differentiating olfactory neurons [80] and other neurons scattered in several brain regions [52]. Hence, these earlier reports and the present results demonstrate that iNOS can be constitutively expressed in different CNS cell types, including microglia, during development and adulthood.

NO synthesized by the action of iNOS in amoeboid microglia would play some physiological role in the normal developing retina. It has been proposed that NO produced by non-microglial cell types in the developing chick retina [73], [75] participates in various functions, including regulation of synaptogenesis, refinement of neural circuits, and control over the arrest of neural cell proliferation at the start of the differentiation process [66], [81]–[83]. NO released by microglia in the developing quail retina might also contribute to these functions *via* paracrine mechanisms. In addition, naturally-occurring neuronal death observed in the quail embryo retina from E7 onward [62] coincides in time with the stronger iNOS immunolabeling of amoeboid microglial cells. Therefore, microglial NO may be related to neuronal death, and *in vitro* studies have shown that microglia can induce neuronal death by a mechanism involving NO [22], [23], [84]. The apoptotic effects of microglial NO may be mediated by peroxynitrite, which is produced by the oxidation of NO with superoxide [27], [33]. It has been demonstrated that naturally-occurring neuronal death is mediated by superoxide production in the developing mouse cerebellum and hippocampus [85], [86]. Hence, it is not unreasonable to conjecture that peroxynitrite produced by the simultaneous release of NO and superoxide in amoeboid microglial cells would play a role in the mechanism of naturally-occurring neuronal death in the developing quail retina. However, it cannot be ruled out that NO also favors neuronal survival, because *in vitro* studies have demonstrated its participation in the inhibition of the apoptotic elimination of embryonic neurons [30], [87]. In fact, it has been suggested that NO has opposite effects on neuronal death depending on its concentration [28], [37]. Microglial NO might be also involved in the phagocytosis of dead neuron debris, given the recent observation by Kakita et al. [88] that NO production is a key regulator of microglial phagocytosis.

Besides its possible paracrine effects, microglial NO might have autocrine effects on the microglial cells themselves and play a role in their migration and proliferation. Microglial migration occurs in the quail embryo retina from E7 onward [60]–[62], coinciding in time with the stronger iNOS immunolabeling of microglial cells reported here. A hypothetical relationship between NO and microglial migration is supported by findings on the role of this molecule in the migration of other cell types, such as endothelial cells [68], [89], [90] and cerebellar neurons [91]. The proliferation of microglia also coincides in time with their stronger iNOS immunolabeling, because microglial cells undergo mitosis simultaneously with their migratory activity [92], and mitosis regulation may be related to NO production. In this respect, it has been suggested that NO is involved in microglial proliferation in the injured prenatal rat brain [46]. Nevertheless, the chronological coincidence between two developmental processes does not prove a causal relationship, and further studies are required to elucidate the possible role of NO in microglial migration and proliferation.

According to the present findings, iNOS immunolabeling is strong in amoeboid microglia of E8 quail embryo retina and continues to be present in ramifying and ramified microglia in more advanced developmental stages and adulthood, although at a much lower intensity. In general, iNOS immunolabeling is stronger in microglial cells migrating tangentially in the vitreal part of the retina and weaker in radially-migrating microglia, coinciding with the start of ramification [63], suggesting a role for NO in the tangential migration of microglia. The weak iNOS immunolabeling in ramified microglia of the adult quail retina implies that iNOS expression is downregulated but continues to be present, as corroborated by our RT-PCR results. The role of NO produced by microglial iNOS in the adult retina is not known and warrants further investigation, although it may contribute to the same NO functions as those of the nNOS expressed in a variety of retinal neurons [76], [77].

### LPS-induced increased activation of amoeboid microglia

iNOS is considered to be a microglial activation marker [93]; therefore, its expression in amoeboid microglia of quail embryo retina supports a certain degree of activation of these cells during normal development. In a strict sense, the concept of microglial activation is reserved for the defensive reaction of ramified microglia against pathological stimuli in the adult CNS. It is characterized by a change from a ramified morphology to a macrophage-like rounded appearance, with increased proliferation, upregulated expression of some surface receptors, and the production of various factors, including growth factors, cytokines (e.g., IL-1β, TNF-α and IFN-γ), NO, and ROS [14], [94], [95]. These factors operate in the microenvironment around activated microglia to restore CNS parenchymal homeostasis [11]. Microglial activation in the adult brain has been considered as almost a retracing in reverse of events observed during brain development [96]. Therefore, in a wider sense, the term activation has also been applied to immature amoeboid microglia during normal CNS development [39]–[41], [97]. This amoeboid microglia activation is triggered by non-pathological signals in the normal developing CNS microenvironment, which stimulate the production of similar factors to those released by activated microglia in the pathological adult CNS, such as cytokines, NO, and ROS. These factors have a role in normal CNS development, contributing to various developmental processes, including neurogenesis and oligodendrogenesis [97], promotion of neuronal survival [98], neuronal commitment to a death fate [99], and execution of engulfment-mediated neuronal death [85], [86]. Microglial activation in the context of normal developmental processes has been referred to as constitutive activation [100], which induces the release of factors at appropriate concentrations for specific developmental functions. Moreover, activated amoeboid microglia in the developing CNS can be additionally stimulated by pathological stimuli or nerve injury, inducing a greater activation degree (over-activation) that gives rise to the massive production of cytokines and ROS, resulting in alterations of normal development. In this respect, the present results show that LPS treatment of *in vitro* cultured E8 retina explants induces iNOS upregulation in amoeboid microglia, as demonstrated by anti-iNOS immunolabeling, western-blot, and RT-PCR, suggesting that their activation level increases in response to LPS, a classic stimulant of microglial activation and inductor of iNOS that is frequently used in *in vitro* [19], [50], [70], [84], [101], [102] and *in vivo* [52], [103]–[105] studies.

The increased activation of microglia in LPS-treated E8 retina explants was verified by analysis of the morphology of microglial cells, following previously reported criteria [38], [98]. The cell rounding index was significantly higher in microglia after LPS treatment, compatible with increased activation, and the relative area of the lysosomal compartment was significantly higher in LPS-treated *versus* control explants. According to these findings, microglia, which show a baseline activation degree in non-treated E8 retina explants, increase their activation level in response to LPS treatment, upregulating their iNOS expression and NO production.
